# Bringing Nitric Oxide to the Molybdenum World—A Personal Perspective

**DOI:** 10.3390/molecules28155819

**Published:** 2023-08-02

**Authors:** Luisa B. Maia

**Affiliations:** LAQV, REQUIMTE, Department of Chemistry, NOVA School of Science and Technology (FCT NOVA), 2829-516 Caparica, Portugal; luisa.maia@fct.unl.pt

**Keywords:** xanthine oxidase, aldehyde oxidase, molybdenum enzyme, nitrite, hypoxia, nitric oxide, cell signalling, moonlighting

## Abstract

Molybdenum-containing enzymes of the xanthine oxidase (XO) family are well known to catalyse oxygen atom transfer reactions, with the great majority of the characterised enzymes catalysing the insertion of an oxygen atom into the substrate. Although some family members are known to catalyse the “reverse” reaction, the capability to abstract an oxygen atom from the substrate molecule is not generally recognised for these enzymes. Hence, it was with surprise and scepticism that the “molybdenum community” noticed the reports on the mammalian XO capability to catalyse the oxygen atom abstraction of nitrite to form nitric oxide (NO). The lack of precedent for a molybdenum- (or tungsten) containing nitrite reductase on the nitrogen biogeochemical cycle contributed also to the scepticism. It took several kinetic, spectroscopic and mechanistic studies on enzymes of the XO family and also of sulfite oxidase and DMSO reductase families to finally have wide recognition of the molybdoenzymes’ ability to form NO from nitrite. Herein, integrated in a collection of “personal views” edited by Professor Ralf Mendel, is an overview of my personal journey on the XO and aldehyde oxidase-catalysed nitrite reduction to NO. The main research findings and the path followed to establish XO and AO as competent nitrite reductases are reviewed. The evidence suggesting that these enzymes are probable players of the mammalian NO metabolism is also discussed.

## 1. Context—I: The Molybdenum Side

Molybdenum is essential to the great majority of organisms [1,2,3,4], from archaea and bacteria to higher plants and mammals, being present in the active site of enzymes that catalyse redox reactions targeting nitrogen, sulfur and carbon atoms of key metabolites [5,6,7,8]. Molybdenum-containing enzymes are responsible for the biological handling of dinitrogen (*Nitrogenase*, Equation (1)), nitrate (*Nitrate reductase* (NaR), Equation (2)), sulfite (*Sulfite oxidase* (SO), Equation (3)), dimethylsulfoxide (dimethylsulfoxide reductase (DMSOR), Equation (4)), aldehydes (*Aldehyde oxidase* (AO), Equation (5)), xanthine (*Xanthine oxidase* (XO), Equation (6)) and carbon dioxide (*Carbon dioxide dehydrogenase*, Equation (7), and *Formate dehydrogenase*, Equation (8)), just to mention a few examples from the more than 50 enzymes presently known.

N≡N + 8H^+^ + 8e^−^ + 16MgATP → 2NH_3_ + H_2_ + 16MgADP + 16P_i_ (1)

ONO_2_^−^ + 2e^−^ + 2H^+^ → NO_2_^−^ + H_2_O (2)

SO_2_^2−^ + H_2_O → OSO_2_^2−^ + 2e^−^ + 2H^+^ (3)

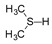
 + 2e^−^ + 2H^+^ → 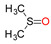 + H_2_O(4)

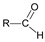
 + H_2_O → 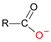 (H^+^) + 2e^−^ + 2H^+^(5)

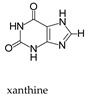
 + H_2_O → 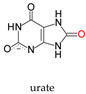 (H^+^) + 2e^−^ + 2H^+^(6)

CO + H_2_O → OCO + 2e^−^ + 2H^+^ (7)

HCOO^−^ → CO_2_ + 2e^−^ + H^+^ (8)

**Figure 1:**
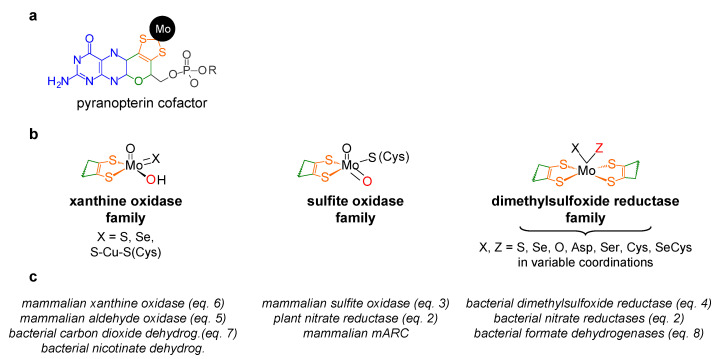
Structures of the molybdenum centres of the three families of molybdoenzymes. (**a**) Pyranopterin cofactor structure. The molecule is formed by pyrano (green)-pterin (blue)-dithiolene (orange) moieties. In eukaryotes, the cofactor is found in the simplest monophosphate form (R is a hydrogen atom; in gray), while in prokaryotes, it is most often found esterified with several nucleotides (R can be one cytidine monophosphate, guanosine monophosphate or adenosine monophosphate). (**b**) Structures of the molybdenum centres, in the oxidised form, of the three families of molybdoenzymes. For simplicity, only the *cis*-dithiolene group of the pyranopterin cofactor is represented. The labile oxygen position is indicated in red. It should be noted that the XO family member carbon monoxide dehydrogenase (X = S-Cu-S(Cys)) is suggested to harbour a labile Mo=O group and not Mo-OH. (**c**) Selected examples of enzymes from each family (mARC, mitochondrial amidoxime reducing component).

With the single (as far as is presently known) exception of nitrogenase [9,10,11,12], molybdenum is found coordinated by the *cis*-dithiolene group (–S–C=C–S–) of one or two molecules of a pyranopterin cofactor (Figure 1). In a parallel situation to the haem ring, this unique cofactor is not an “innocent scaffold” and it is considered to be co-responsible to modulate the active site reactivity, besides acting as a “wire” to conduct the electrons to, or from, the other redox-active centres of the enzyme (intramolecular electron transfer, when this is the case) [13,14,15,16,17,18,19,20]. In addition to the pyranopterin cofactor, the molybdenum ion is coordinated by oxygen and/or sulfur and/or selenium terminal atoms and/or by enzyme-derived amino acid residues (Figure 1), which are also expected to have key roles in catalysis (although their individual roles are not yet understood in many molybdoenzymes). To introduce order in such a diversity of molybdenum compositions and (try to) rationalise the catalytic features, the molybdoenzymes were classified into three large families, denominated after one benchmark enzyme, as indicated in Figure 1 [5]: XO family, SO family and DMSOR family.

**Figure 2:**
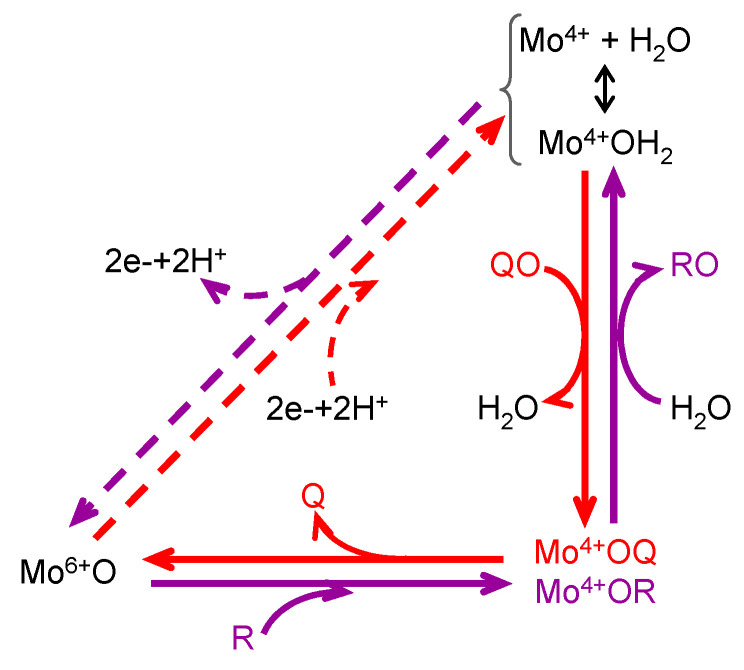
General mechanism of molybdoenzymes-catalysed OAT. The molybdenum role as electron acceptor/donor and as direct oxygen donor to substrate/oxygen acceptor from product is highlighted. OAT-I is represented in violet; e.g., for SO (Equation (3)), R would be sulfite and RO is sulfate; reduction of the enzyme physiological partner/regeneration of the enzyme active site is represented in dashes. OAT-A is represented in red; e.g., for NaR (Equation (2)), QO would be nitrate and Q is nitrite; oxidation of the enzyme physiological partner/regeneration of the enzyme active site is represented in dashes. It should be noted that catalysis can take place without the occurrence of the dashed reduction/oxidation.

These molybdenum centres have been exploited by living organisms to carry out different reaction types [5,6,7,8], many of which are oxygen atom transfer reactions (OAT), where an oxygen atom is transferred from water to product -*oxygen atom insertion* (OAT-I) (Figure 2, violet arrows)- or from substrate to water -*oxygen atom abstraction* (OAT-A) (Figure 2, red arrows).

**Figure 3:**
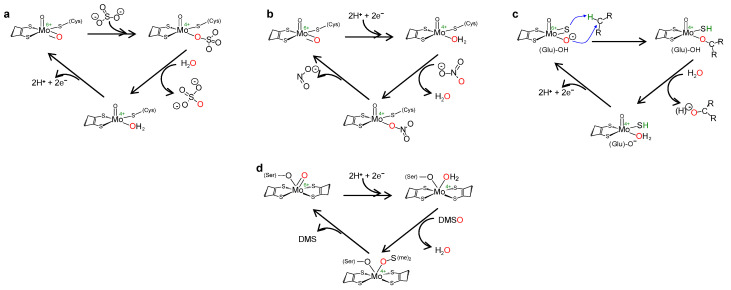
Simplified mechanism of OAT catalysed by eukaryotic SO (**a**), NaR (**b**), XO (**c**) and prokaryotic DMSOR (**d**). See text for details.

The reaction mechanism of molybdoenzymes-catalysed OAT-I and OAT-A is presently well established and can be illustrated with the eukaryotic SO (Equation (3)) and NaR (Equation (2)) catalytic cycles (two enzymes from the SO family). Briefly, in SO [21,22,23,24,25,26,27,28,29,30,31,32,33,34] (Figure 3a), catalysis is initiated at the oxidised molybdenum centre, where its equatorial labile oxido group (Mo^6+^=O_equatorial_) is (nucleophilically) attacked by the sulfite lone-pair of electrons, resulting in the formation of a reduced, covalent Mo^4+^-O-SO_3_ intermediate. After cleavage of the Mo-O(substrate)_equatorial_ bond, sulfate is released (oxidation half-reaction). The Mo^4+^-OH_(2)_ centre thus formed is then re-oxidised by two electrons (with the eventual reduction of the SO physiological partner—reduction half-reaction) to regenerate the initial Mo^6+^=O centre. The mechanism of OAT-A of NaR is suggested to be the reverse of the SO one (Figure 3b) [35,36,37,38,39,40,41], starting with the reduction of the molybdenum centre (two electrons provided by the NaR physiological partner—oxidation half-reaction). Nitrate binding yields the covalent intermediate Mo^4+^-O-NO_2_, which, after cleavage of the O-N bond and release of nitrite (reduction half-reaction), regenerates the Mo^6+^=O core. Hence, in a simplistic way, the molybdenum role in OAT is to accept/donate the necessary electrons for these redox reactions and to act as the direct oxygen donor to substrate/oxygen acceptor from product (Figure 2).

This general OAT mechanism (Figure 2) is suggested to be followed by other molybdoenzymes, from other families, but with some variations imposed by the respective substrate nature. This is the case of the XO-catalysed xanthine hydroxylation to urate (Equation (6); OAT-I) a reaction more “complex” than the “simple” OAT of SO and NaR, because it requires the cleavage of the stable C(8)-H bond of xanthine for the oxygen to be inserted. To catalyse the xanthine hydroxylation, the molybdenum active site of XO holds an equatorial labile oxido group that is the direct source of the oxygen atom to be inserted into the xanthine C(8) (in parallel to SO). However, in the place of the S-coordinated cysteine residue of SO, XO active site harbours a terminal sulfido group to accept the xanthine H(8) and make its labile oxide group capable of carrying out a nucleophilic attack (instead of undergo a nucleophilic attack as in SO). Briefly, XO catalysis (Figure 3c) [42,43,44,45,46,47,48,49,50,51,52,53,54,55,56] is initiated with the activation of the equatorial labile oxido group by a conserved glutamate residue, to form the catalytically competent Mo^6+^(=S)-O^−^ centre. This makes a nucleophilic attack into xanthine C(8), while the sulfido group accepts the xanthine H(8) as a hydride, leading to the formation of a reduced, covalent intermediate Mo^4+^-O-C-R(-SH) (where R represents the remainder of the xanthine molecule). After hydrolysis of the Mo-O(xanthine)_equatorial_ bond, urate is released (oxidation half-reaction) and the Mo^4+^(SH)-OH_(2)_ centre formed is re-oxidised by two electrons (with the eventual reduction of the XO physiological partner—reduction half-reaction) to regenerate the initial Mo^6+^(=S)-O^−^ centre.

The enzymes from the DMSOR family, in spite of having its active site molybdenum ion coordinated by two molecules of the pyranopterin cofactor (Figure 1), are also suggested to follow the same general OAT mechanism (Figure 2), as illustrated, for example, by bacterial DMSOR itself (Figure 3d) [57,58,59,60,61,62,63,64,65,66,67] or bacterial NaRs [64,66,67,68,69,70,71,72,73,74,75,76,77,78,79,80,81]. Bacterial nitrite oxidoreductases, that catalyse the oxidation of nitrite to nitrate (NaR reverse reaction), also follow a typical OAT-A mechanism [82,83,84,85,86,87,88,89,90,91,92]. Noteworthy, to date, no parallel molybdenum-dependent nitrite reductase has been identified.

## 2. Context—II: The Nitric Oxide Side

**Figure 4:**
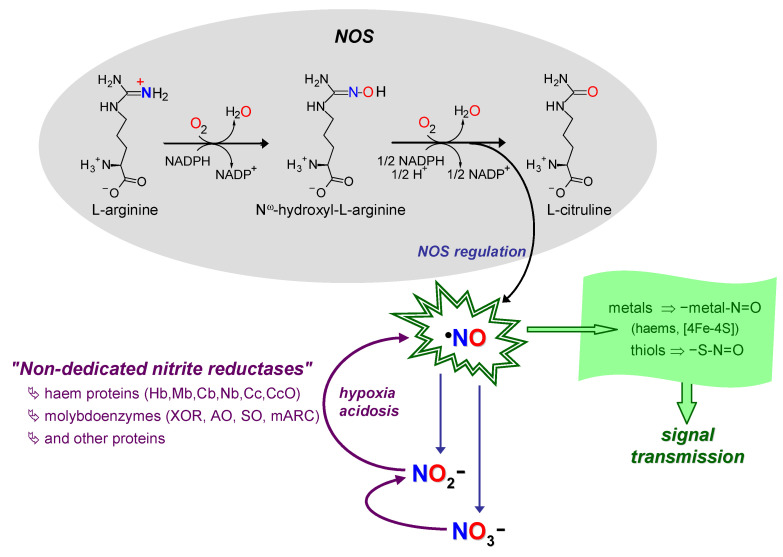
Schematic summary of NO formation–function–extinction. NO formation through NOS and nitrate and nitrite-dependent pathways (black arrows/gray shadowed area and violet arrows, respectively). NO consumption in signalling/function (arrow and shadowed area in green). NO extinction in reactions to limit its toxicity and control the signal specificity (oxidation to nitrate and nitrite; indigo arrows). Hb, haemoglobin; Mb, myoglobin; Cb, cytoglobin; Nb, neuroglobin; Cc, cytochrome *c*; CcO, Cc oxidase. See text for details. Adapted from [111] with permission.

Nitric oxide radical (^∙^NO, herein abbreviated as NO) is a signalling molecule involved in several physiological processes and, consequently, its deficit or excess is associated with many pathological conditions. In humans, NO is produced, in a tightly regulated manner, by three isoforms of NO synthase (NOS; neuronal, endothelial and inducible NOS), using L-arginine and dioxygen as the source of the NO nitrogen and oxygen atoms, respectively (Equation in Figure 4) [93,94,95,96]. The “NO signal” is transmitted intra- and extracellularly mainly through post-translational modifications of transition metal centres and thiols. The targets are mostly labile [4Fe-4S] centres and hemes (as is the case of the well-known activation of guanylate cyclase) and cysteine residues, that are converted into the respective nitrosyl (–metal-N=O) and nitrosothiol (-S-N=O) derivates. In addition, to limit the NO toxicity and ensure the fast “on/off signal” response, the NO life time is controlled through its rapid oxidation to nitrate (by reaction with oxy-hemoglobin and oxy-myoglobin [97,98,99,100,101,102,103,104]) and also to nitrite (by ceruloplasmin [105], cytochrome *c* oxidase [98,106,107] or dioxygen [108,109,110]) (Figure 4).

This NO metabolic flux, formation–function–extinction (Figure 4), where nitrate and nitrite are considered end-products, with no physiological function, was firmly established by the end of the 20th century, when new ideas emerged. It began to become clear that nitrite can be reduced back to NO under anoxic conditions (Equation (9)) and exert a cytoprotective role during in vivo ischaemia and other pathological conditions [112,113,114,115,116,117,118,119,120,121,122,123,124,125,126,127,128,129,130,131,132,133,134,135,136,137,138,139,140,141,142,143,144,145,146]. In accordance, nitrite began to stop being seen as a useless end-metabolite, to become a “storage form” of NO. This new, alternative, NO source would be key to maintain the NO formation and ensure cell functioning under conditions of hypoxia/anoxia, precisely when the dioxygen-dependent NOS activity is impaired (Equation in Figure 4) and a “rescue” pathway is needed.

ONO^−^ + 1e^−^ + 2H^+^ → ^∙^NO + H_2_O (9)

At the same time, numerous studies began to be carried out to identify the pathway(s) responsible for nitrite reduction in mammals (humans), and, later, also in higher plants. The quest did not lead to the identification of any mammalian NO-forming nitrite reductase. Instead, the mammalian nitrite reduction activity was assigned to (already well-known) proteins that are present in cells to perform other (well-known) functions [147,148,149,150,151,152,153,154,155,156,157,158,159,160,161,162,163,164,165,166,167,168,169,170,171,172,173,174,175,176], including, at first, mainly the haem proteins haemoglobin (Hb) and myoglobin (Mb) and the molybdoenzyme XO (Figure 4). However, like all new ideas, the ability of those and other proteins to use nitrite to form and deliver NO faced severe criticism. Many authors questioned the physiological relevance of the NO fluxes generated from the physiologically available (very low) nitrite and called attention to the inhibition by other metabolites. For the “haem community”, the hurdle was the NO delivery, because of the (for long) known ability of haems to easily bind NO and hardly dissociate it. With XO, the main problem was its ability to form NO. The reaction feasibility was controversial, because XO is a well-established OAT-I enzyme (Equation (6)), rather than the OAT-A catalyst needed to reduce nitrite to NO (Equation (9)), and dioxygen was believed to completely inhibit the reaction.

## 3. How it Began…

**Figure 5:**
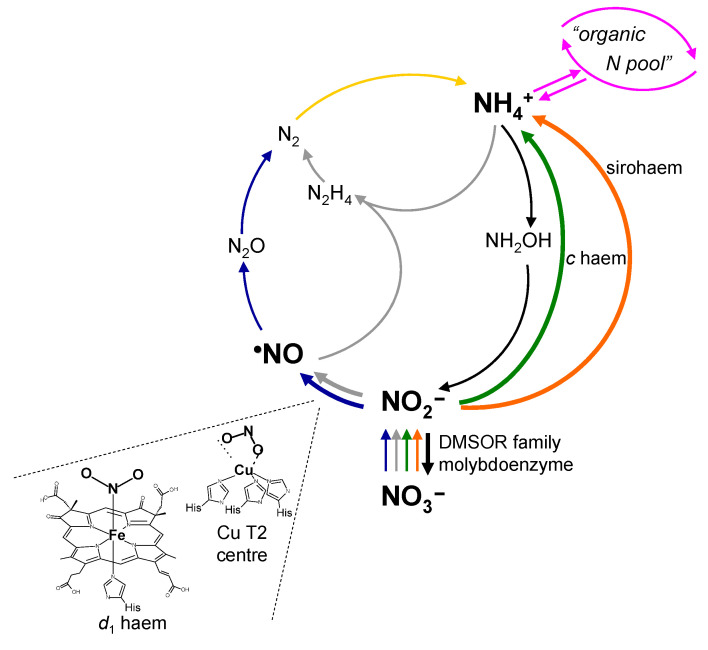
Overview of the biogeochemical cycle of nitrogen, highlighting the steps involving nitrite reduction and oxidation. In the “respiratory” pathways denitrification (blue arrows) and anaerobic ammonium oxidation (AnAmmOx; gray arrows), nitrite is reduced to NO by copper type 2 centre or *d*_1_ haem-dependent nitrite reductases (the active site metal centres structures are also shown). On the other hand, nitrite is reduced to ammonium in both assimilatory ammonification (nitrogen assimilatory pathway; orange arrows) and dissimilatory nitrate reduction to ammonium (“respiratory” pathway; green arrows), by nitrite reductases dependent on sirohaem and *c* haem, respectively. During nitrification or complete ammonium oxidation (ComAmmOx; two “respiratory” pathways; black arrows), nitrite is oxidised to nitrate by a molybdoenzyme from the DMSOR family, nitrite oxidoreductase. Although not involving nitrite, the dinitrogen fixation (nitrogen assimilatory pathway, catalysed by nitrogenases; yellow arrow) and the “organic nitrogen pool” (where the ammonium fluxes are controlled by glutamine synthase, glutamate synthase and glutamate dehydrogenase; pink arrows) are also represented. Adapted from [178] with permission.

During my PhD, working with mammalian liver XO, its dehydrogenase form (XD) and AO on “free radicals” and their pathological implications, I had the chance to learn about the biological versatility of molybdoenzymes and their fascinating chemistry (briefly reviewed above). By that time, the nitrite reduction to NO by XO had already been described and contested by several authors of the “more medical-oriented community”, who argued that (i) it would need a high, non-physiological concentration of nitrite to promote a significant generation of NO and (ii) it should be completely inhibited by dioxygen (the physiological oxidising substrate of XO and AO). Discussing the state-of-the-art on mammalian molybdoenzymes with my (already then) good friend José Moura, I realised that the “molybdenum community” would also have reservations and would find the XO ability to reduce nitrite questionable. The lack of precedent for a molybdenum- (or tungsten) containing nitrite reductase on the nitrogen biogeochemical cycle (Figure 5) [177,178,179,180,181,182,183] also contributed to the general scepticism about the XO, or other molybdoenzyme, nitrite reductase activity.

Knowing that molybdenum centres are excellent “oxygen atom exchangers” [184,185,186,187,188,189,190,191,192,193,194] and that molybdoenzymes are very versatile, I did not quite understand the reason for so many doubts. At least from a purely chemical perspective (that is, in vitro), the reaction seemed to me worthy of being studied! Its physiological occurrence and relevance (the in vivo) was a completely different story and I shared other authors’ concerns, particularly regarding dioxygen inhibition. Therefore, when I finished my PhD and José Moura invited me to join his research group (Bioinorganic Lab, FCT NOVA, Lisboa, Portugal), I immediately accepted the challenge and set out to answer three key questions:

(1) are mammalian XO/XD and AO able to generate NO? with what magnitude and kinetics? (aiming to confirm the enzymes’ ability to reduce nitrite to NO and study the kinetics and magnitude of NO generation);
(2) how is it possible for XO (and similar enzymes) to catalyse an OAT-A reaction? (aiming to establish the reaction mechanism of nitrite-derived NO formation);
(3) if no other organism is known to use a “true” molybdenum-dependent nitrite reductase to reduce nitrite to NO, why would a mammalian cell be able to do so? (aiming to assess the reaction physiological feasibility and significance).

## 4. My Journey on the Molybdenum-Dependent, “Non-Dedicated” Nitrite Reductases

I was very well received in both José Moura and Isabel Moura research groups and I have to thank them and their collaborators for all the doubts and questions they raised about the molybdenum-dependent nitrite reduction. They pushed me to go further and also to learn more about the enzymes of the nitrogen biogeochemical cycle, which was essential to put the molybdenum reactivity into the context of the chemical strategies biology uses to handle nitrite.

### 4.1. Are Mammalian XO/XD and AO Able to Generate NO?

First and foremost, it was mandatory to confirm, beyond any doubt, that nitrite is reduced by XO/XD and AO and that NO is the product of the reaction and it is released from the enzymes (necessary condition for NO to be physiologically available to exert its function). Most studies were carried out with XO/XD and AO purified from rat liver, using a simple procedure that I had implemented during my PhD studies [195]. Small quantities of real human enzymes were also purified to corroborate all main results obtained with the rat enzymes. The bacterial aldehyde oxidoreductase (AOR), an enzyme structurally and functionally very similar to the mammalian counterparts, identified for the first time by José Moura [196,197,198,199,200,201], was also studied to enlarge the sampling of potential molybdenum-dependent nitrite reductases.

**Figure 6:**
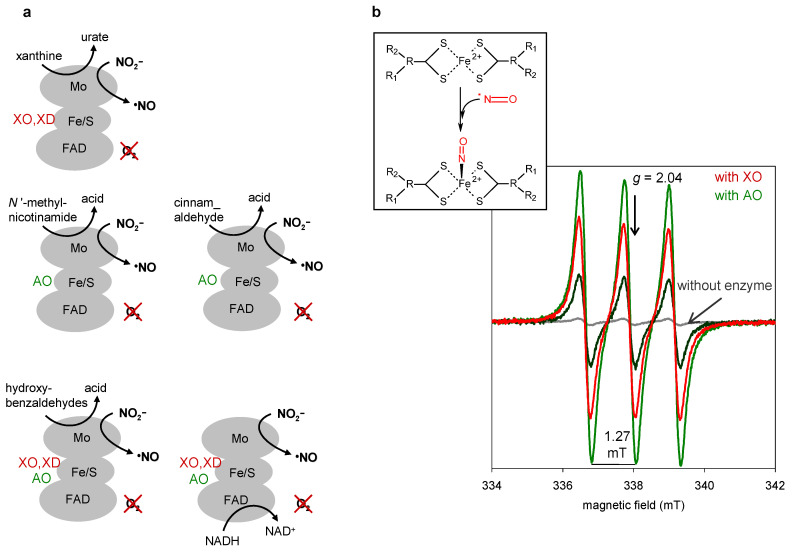
Experimental evidences of the XO/XD and AO-catalysed nitrite reduction to NO. (**a**) Schematic representation of the kinetic assays performed with different reducing substrates (with different chemical structures), in the presence of nitrite, under anaerobic conditions, in which the catalytic oxidation of the reducing substrate was used as proof of the enzymes’ ability to catalytically reduce nitrite. Also illustrated are the enzymes’ redox active-centres involved in each reaction (molybdenum centre (Mo), two [2Fe-2S] (Fe/S) and FAD). See text for details. (**b**) Representative EPR spectra, using the spin-trap (MGD)_2_-Fe, represented in the structure on top, showing the characteristic triplet signal (*g* ≈ 2.04, *A*^N^ = 1.27 mT) arising from (MGD)_2_-Fe-NO. See text for details.

Showing the enzymes’ ability to reduce nitrite was not difficult. The observation of catalytic oxidation of diverse XO/XD and AO reducing substrates, in the absence of any oxidising substrate (assays under anaerobic conditions), but presence of nitrite, was the proof that nitrite was being concomitantly catalytically reduced (Figure 6a) (if nitrite was not acting as a catalytic co-substrate, then there would be no oxidation of the oxidising substrate or, at the most, there would be only single-turnover oxidation) [202,203]. Moreover and most important, the reaction rate was found to be dependent on the nitrite concentration (besides the enzyme and reducing substrate concentrations).

That set of kinetic assays also demonstrated that the nitrite reduction is independent of the nature of the reducing substrate assayed [203,204]. The reaction (in vitro) is achievable with substrates having different chemical structures and reacting at the different enzyme sites (molybdenum centre or FAD; Figure 6a), which suggests that the nitrite reduction should be feasible with the physiologically available reducers in vivo.

The nature of the product of nitrite reduction was a more complex question to address. The biological reduction of nitrite to NO was already known, but only in prokaryotic “respiratory” pathways, where it is catalysed by copper-containing or *d*_1_ haem-containing nitrite reductases (Figure 5) [178,180,183,204]. However, nitrite can be also reduced to nitroxyl (NO^−^) or ammonium (NH_4_^+^; Figure 5). To unequivocally confirm the nitrite reduction to NO, a radical species, electron paramagnetic resonance (EPR) spectroscopy was key. The EPR spectrum provides quantitative and qualitative information regarding the nature, structure and environment of the paramagnetic species and, therefore, unquestionable information about the concentration and identity of the radical species. Therefore, we, like other authors [147,148,150,151,152,160,161], turned to EPR and, using the spin-trap iron-*N*-methyl-D-glucamine dithiocarbamate ((MGD)_2_-Fe) [205], successfully demonstrated the formation of a mononitrosyl-iron complex (MGD)_2_-Fe-NO), with its clear-cut triplet signal (*g* ≈ 2.04, *A*^N^ = 1.27 mT; Figure 6b), and, in this way, the XO/XD and AO-catalysed NO generation [202,203]. Parallel assays with NO scavengers (that do not inhibit these enzymes) gave further support to the enzymes’ ability to release NO. We complemented these EPR assays with detailed polarographic studies, employing a NO-selective electrode, while other authors used chemiluminescence NO detection. Collectively, these three independent methodologies were essential to prove that it is NO that is being formed and released by these enzymes.

We went further and demonstrated that the enzymes are not inactivated or inhibited by NO [202,203]. NO can react with cysteine residues and metal centres (in the case of XO/XD and AO, their molybdenum and iron/sulfur centres and XD surface cysteine residues), affecting the enzymes’ activity. In addition, because the nitrite reduction to NO is a one-electron reduction process (Equation (9)), there was also the concern that the molybdenum centre got arrested in a Mo^5+^ oxidation state (Mo^4+^ → Mo^5+^) and, thus, unable to be regenerated back to the catalytically competent oxidised form (Mo^6+^; Figure 2 and Figure 3). Analysis of the reaction time courses obtained with NO-selective electrode (that exhibit the expected exponential shape) confirmed the absence of inactivation or inhibition events or of non-enzymatic processes. Additional experimental evidence was provided by the EPR spectra of the enzymes’ redox centres, which exhibit the characteristic EPR signals during and after the nitrite reduction catalysis [202,203,206,207].

The above reaction time courses were also analysed to quantify the total NO generated. The curves showed that approximately two NO molecules are formed per reducing substrate molecule consumed [202,203], which can be taken as additional evidence of the catalytic competence of XO and AO to generate NO (note that XO and AO oxidise their reducing substrates by two electrons (Equations (5) and (6)), while the nitrite reduction to NO is a one-electron process (Equation (9)), so, a 1:2 relation would be expected).

### 4.2. Kinetics and Magnitude of XO/XD- and AO-Generated NO

Once the enzymes’ ability to reduce nitrite to NO was confirmed, it was necessary to assess the kinetics and magnitude of the nitrite reduction by each enzyme, to have a glance at how relevant the NO generation by each enzyme could be in vivo.

At first, the kinetic characterisation was performed under anaerobic conditions, using similar nitrite and enzyme concentrations and reducing power (i.e., a reducing substrate with a similar rate of reduction of all enzymes) [202,203]. Reflecting the similarity of their molybdenum centres, it was found that XO, XD and AO behave very similarly. Moreover, rat and human liver enzymes displayed comparable kinetic parameters [203], which validated the utilisation of the rat liver enzymes (whose tissue is easier to obtain) as a suitable model of the human counterparts.

**Figure 7:**
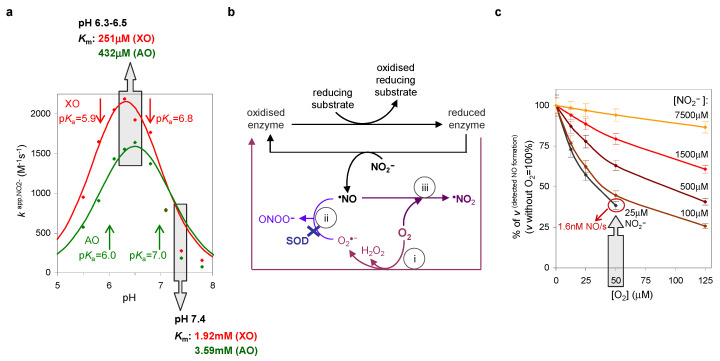
pH (**a**) and dioxygen (**c**) effects on the kinetic parameters of XO- and AO-catalysed nitrite reduction to NO. (**b**) Schematic representation of how dioxygen decreases the amount of NO formed (i) and NO available to exert its in vivo actions and also to be detected in the Lab (ii) and (iii). In the presence of superoxide dismutase (SOD), the NO consumption by superoxide radical anion (ii) is abolished (as represented by X) and the formation of peroxynitrite also prevented. See text for details. Adapted from [111] with permission.

Noteworthy, the XO- and AO-catalysed NO formation was found to be pH-dependent, being greatly favoured (accelerated) at acidic pH values. Comparatively to the “normal” pH of 7.4, the nitrite reduction rate constants were found to increase almost 10 times when the pH is lowered to 6.3 ($k^{\mathrm{app},}{}^{{\mathrm{NO}}_{2}{}^{-}}$ of 2.20 × 10^3^ M^−1^ s^−1^ (XO) and 1.64 × 10^3^ M^−1^ s^−1^ (AO)), while the $K_{m}{}^{NO_{2}{}^{-}}$ values significantly decrease (251 μM (XO) and 432 μM (AO)) (Figure 7a) [203]. These results were very interesting for two reasons. First, because upon an ischaemic event, or any other condition of hypoxia/anoxia, the cellular pH can drop to values as low as 6.0–5.5 (acidosis) [208,209,210]. Therefore, under hypoxia/anoxia, when the activity of NOS would become compromised, the characteristic acidic conditions could accelerate the NO generation by these enzymes, as a salvage pathway. 

Second, the lower $K_{m}{}^{{\mathrm{NO}}_{2}{}^{-}}$ values indicate that less nitrite is required to promote the NO formation. The need for a millimolar concentration of nitrite (order of the $K_{m}{}^{{\mathrm{NO}}_{2}{}^{-}}$ values at pH 7.4), when the physiologically available nitrite is much lower (<20 μM [211,212,213]), was one of the strongest criticisms raised against the involvement of these enzymes in NO generation. However, the generation of a signalling molecule like NO should not occur as a burst of micromolar NO (such burst would lead to an “indefinitely on” signal and result in an overproduction of highly deleterious nitrogen reactive species). But our results showed that, at the acidic pH characteristic of a hypoxia/anoxia, when an alternative NO generation pathway is needed, XO and AO affinity towards nitrite is enhanced and the available nitrite could elicit the formation of physiologically meaningful NO. For example, at pH 6.3, 10 μM nitrite and 30 μg enzyme/mL (to simulate 30 μg enzyme/g tissue) catalyse the formation of 1.6–2.2 nM NO/s [203], rates that compare well with the 1 nM/s described for the constitutive NOS [214].

After the detailed kinetic characterisation under anaerobic conditions, the effect of dioxygen on the NO formation by XO/XD and AO had to be studied. Yet, assessing the effect of dioxygen is not a trivial task, quite the opposite, and the assays took much longer than initially anticipated. Dioxygen is efficiently reduced by these enzymes (it is the physiological oxidising substrate of XO and AO) and, by consuming the electrons needed to reduce nitrite, it should act as a strong competitive inhibitor of the NO formation, thus decreasing the total amount of NO formed (Figure 7b—reaction (i)). For the same reason, the inhibition of the XD-dependent NO formation by NAD^+^ should have also been considered, but that study was not yet undertaken (neither by myself nor by any other author, as far as I know). On the other hand, the superoxide anion formed during the dioxygen reduction by XO and AO should promptly react with NO to yield peroxynitrite and, in this way, decrease the amount of NO available to exert its in vivo actions and also to be detected in the Lab (Figure 7b—reaction (ii)) [203]. This indirect consumption of NO by dioxygen has been wrongly interpreted as an inhibition of NO formation and has contributed to the inconsistencies found in the literature. In addition, dioxygen can also directly consume (react with) NO, resulting in the formation of ^∙^NO_2_ (Figure 7b—reaction (iii)).

The NO consumption in the direct reaction with dioxygen (as assessed using deflavo-XO and deflavo-AO, enzyme forms that cannot reduce dioxygen and produce superoxide) was not significant under our experimental conditions and was disregarded [203]. On the contrary, the NO consumption in the reaction with superoxide was considerable, but not total, and it was still possible to detect it in the absence of added superoxide dismutase (SOD) (Figure 7b—reaction (ii)) [203]. These results highlighted the physiological relevance of SOD to achieve a net flux of NO in vivo, as well as to avoid the formation of the highly deleterious peroxynitrite.

To restrict our studies to the dioxygen effect in NO formation, the following assays were carried out in the presence of SOD (this simplified the results analysis by eliminating the NO consumption by superoxide). Even so, the system continued to be quite complex to analyse, but the results very interesting. It was found that the inhibition of NO formation by dioxygen is dependent on the reducing substrate concentration, with a higher reducing power giving rise to lower inhibitions [203]. In accordance, it is expected that the ischaemia-induced accumulation of reducing substrates would decrease the inhibition imposed by dioxygen—once more the results were pointing to hypoxia/anoxia boosting the NO generation by these enzymes. The $K_{i}{{}^{\mathrm{app},}}^{O_{2}}$ values of XO (24 μM) and AO (25 μM) fully supported this idea [203]. These dioxygen values are within the in vivo tissue dioxygen concentrations, going from normoxia (around 50 μM) to hypoxia (>2 μM) [215], which suggests that the cellular dioxygen availability would, in an integrated mode, fine-tune the NO formation by XO and AO. That is, under normoxic conditions, the NO formation by these enzymes would be considerably hindered and it would be NOS to sustain the NO fluxes (no alternative pathway needed); however, as the dioxygen concentration decreases towards hypoxic and anoxic conditions and NOS becomes impaired, the concomitant acidic conditions and accumulation of reducing substrates would accelerate and “fuel” the nitrite reduction and the dioxygen inhibition would be relieved, all resulting in the triggering of NO generation by XO and AO, as a NO “backup” pathway.

Also, and more importantly, the XO- and AO-catalysed NO generation in the presence of dioxygen occurs at rates that can be physiologically meaningful. For example, 1.3–1.9 nM NO/s and 2.0–2.9 nM NO/s can be formed from 25 μM nitrite in the presence of dioxygen 50 μM (normoxia) and 25 μM (hypoxia), respectively [203], values that compare very well with the 1 nM/s described for the constitutive NOS [214]. These results clearly contradict the general belief that there is no NO formation by these enzymes in the presence of dioxygen—the second major criticism against the involvement of these enzymes in NO metabolism.

### 4.3. How Is It Possible for XO (and Similar Enzymes) to Catalyse an OAT-A Reaction?

Molybdoenzymes are well known for their ability to catalyse OAT-A and OAT-I and the respective reaction mechanism is presently well established, as briefly reviewed in Section 1. XO family enzymes, in particular, are commonly associated with hydroxylation reactions (OAT-I; Equations (5) and (6)), but the active site of the “XO type” is certainly able to catalyse the reverse “dehydroxylation” (OAT-A), as exemplified by the bacterial hydroxybenzoyl-CoA reductase (Equation (10)) [216,217,218,219]. The NO-forming nitrite reductase activity of XO/XD and AO is now demonstrating that this versatile family is also able to catalyse the simultaneous OAT-A and OAT-I, during the same catalytic cycle. Yet, given the straightforward reversibility of the OAT mechanism (Figure 2—reaction can take place without the occurrence of the oxidation/reduction in dashes) and the precedent given by the bacterial pyrogallol:phloroglucinol transhydroxylase (a DMSOR family enzyme; Equation (11)) [220], the simultaneous OAT-A and OAT-I should have been anticipated before.

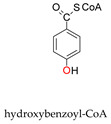
 + 2e− + 2H^+^ → 
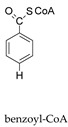
 + H_2_O(10)

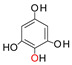
 + 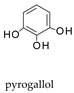 → 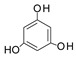 + 
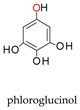
(11)

**Figure 8:**
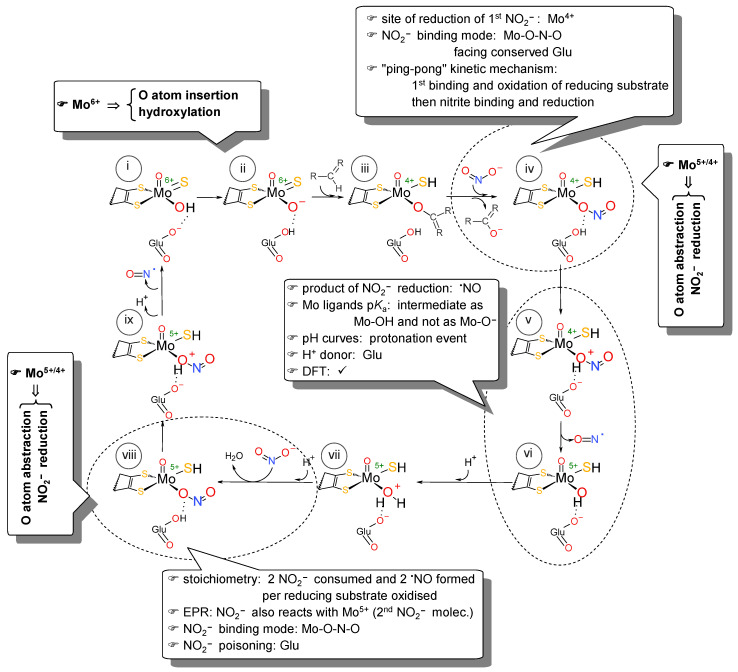
Reaction mechanism proposed for XO- and AO-catalysed nitrite reduction to NO, highlighting the details and evidences supporting each step. After the release of the oxidised-reducing substrate (for example, urate; i→ii→iii), nitrite binds to the reduced molybdenum ion through one of its oxygen atoms (iii→iv). Subsequently, a protonation step, where the nitrite oxygen atom is protonated (v), triggers the homolyitc O-N bond cleavage, resulting in the formation of the first NO molecule and a partially oxidised molybdenum (Mo^5+^) centre (v→vi). The residue responsible for the protonation step is suggested to be the conserved glutamate residue that is essential for the hydroxylation half-reaction (Glu_1261_ in bovine milk XO). The reaction proceeds with the formation of a good leaving group, a water molecule (Mo^5+^–OH_2_; vi→vii), which is subsequently displaced by the second molecule of nitrite (vii→viii). After a second cycle of nitrite reduction/molybdenum oxidation, triggered by another protonation step, the second NO molecule is released (viii→i). The molybdenum is now in a 6+ oxidation state, which would favour the deprotonation of its ligands, and the centre is ready to start a new catalytic cycle. More details in [111,203,206]. Adapted from [111] with permission.

We have proposed the first and currently accepted molecular mechanism of nitrite reduction by molybdoenzymes (Figure 8) [111,202,203,206]. This was based on different experimental evidences that we and others gathered, combining EPR and NO electrode assays, exploring enzyme preparations with different AFR values, deflavo and desulfo forms [150,202,203] and using flavin- and molybdenum-specific inhibitors (DPI (XO and AO), allopurinol, BOF-4272 (XO) [148,150,151,202,203] and ethyleneglycol (AOR) [202]). Attention was also paid to the possible, but ruled out, reaction of NO with the molybdenum sulfido group (that would lead to the enzymes’ inactivation by formation of a molybdenum-nitrosothiol active site) [202,203].

Briefly, after the oxidation of the reducing substrate and release of the hydroxylated product (as described in Section 1; Figure 3c), nitrite would bind to the reduced molybdenum centre through one of its oxygen atoms. Its subsequent reduction should occur through a mechanism parallel to the OAT-A of NaR (Figure 3b), but involving the consecutive reaction of two molecules of nitrite (because nitrite reduction to NO, contrary to nitrate reduction, is a one-electron process). For the protonation steps required to abstract the nitrite oxygen atom, we proposed as proton donor the conserved and well-positioned glutamate residue (the same that is essential to the hydroxylation half-reaction, Glu_1261_ in bovine milk XO; see Section 1). Interestingly, the bacterial copper-containing nitrite reductase also uses an amino acid residue for this role (in that case, an aspartate).

### 4.4. If No Other Organism Is Known to Use a “True” Molybdenum-Dependent Nitrite Reductase to Reduce Nitrite to NO, Why Would a Mammalian Cell Be Able to Do So?

To date, no “true” mammalian nitrite reductase has ever been identified. Yet, numerous (very well-known) proteins were shown (by several authors) to be able to reduce nitrite to NO [147,148,149,150,151,152,153,154,155,156,157,158,159,160,161,162,163,164,165,166,167,168,169,170,171,172,173,174,175,176] and also some protein-independent NO generation pathways were proposed [221,222,223,224,225,226,227,228]. In all those cases, the nitrite reduction to NO was described to occur under hypoxic/anoxic and acidic conditions, but most of those systems were not thoroughly characterised in vitro, ex vivo or in vivo. Presently, as far as I know, only Mb and XO/XD have been demonstrated to be directly involved in the cytoprotective action of nitrite in vivo or ex vivo [115,122,229,230,231]. The ground-breaking work of Webb et al. [115], which isolated a rat heart under ischaemic conditions, demonstrating the NOS-independent, largely XO/XD-dependent NO generation and cardioprotective protection, was inspirational to me. The following contributions of Baker et al. [122] and Baliga et al. [142] were also relevant.

I was (am) well aware of the importance of physiological studies and wanted to make some “forays” into this area. However, the choice of the right biological model is critical and measuring the NO formation in vivo is a very challenging task, with no clear ideal methodology to follow (NO-specific electrodes or chemiluminescence (that measures NO only in the gas phase) only detect NO after cell disruption or after NO has diffused out of the cell/tissue; microelectrodes only solve that problem until a certain scale (μm); non-toxic cell-permeable probes, such as spin-traps (EPR) or fluorescence probes, can still induce alterations on the biological system and the fluorescence probes are not NO-selective in the biological milieu). As no one in our group had experience in physiological studies, we turned to our good friend Lurdes Mira, who had been carrying out several studies of a “more medical nature”. Her team implemented a fluorescent method capable of measuring intracellular NO and exploited the use of different specific inhibitors to assess the individual contributions of a few NO generation pathways (NOS, mitochondrial complex I, XO/XD and AO). Lurdes Mira’s efforts were essential to demonstrate the nitrite-derived, XO/XD- and AO-dependent NO generation in a hypoxia model (23 μM dioxygen) using human epithelial cells from liver carcinoma (HepG2) and human microvascular endothelial cells (HMEC) [203]. Remarkably, XO/XD and AO were found to account for as much as ≈50% of the measured NO in these systems.

## 5. Concluding Remarks

In the early 21st century, a new nitrate-nitrite-NO pathway began to emerge as a physiological alternative to the “classic”, NOS-dependent, pathway and, presently, nitrite is recognised as a relevant NO source for human cell signalling and survival under challenging conditions. In the absence of a “true” nitrite reductase, mammalian cells appear to reduce nitrite to NO using different metalloproteins, present in the cells to accomplish other (well-known) functions. The list of those proteins, that in 2014 we denominated as the “non-dedicated nitrite reductases”, is actually quite long and it includes, among others, several haem proteins and all known mammalian molybdoenzymes, XO/XD, AO, SO and mARC.

Over the past years, I have put a lot of effort into understanding the participation of two molybdoenzymes, XO/XD and AO, in the nitrite-derived NO generation. All the results obtained point to the same conclusion: XO/XD and AO do reduce nitrite and form NO, as would be expected from a purely chemical perspective, as molybdenum centres are known to be excellent “oxygen atom exchangers”. However, acidic and hypoxic conditions are needed to amplify the NO formation (higher reaction rate and magnitude) and make the reaction kinetically relevant in the physiological context. During this time, other authors (as cited above) came along and contributed to show that other molybdoenzymes are also able to catalyse the nitrite reduction, namely SO (mammalian enzyme), mARC (plant and mammalian enzymes) and NaR (plant and bacterial enzymes). All these cases suggest that, in the presence of both one oxo donor and one oxo acceptor, the molybdenum centres of the three enzyme families (Figure 1) would be able to catalyse the OAT between the two and, therefore, this “double oxo transfer” reaction should be possible for substrates other than nitrite (as long as the thermodynamics of the global reaction are favourable).

The physiological relevance of the molybdoenzymes in NO metabolism is still, however, a matter of debate, in particular in humans, and I look forward to future works that shed light on the role of nitrite (and also nitrate) in humans. In any case, I believe that nitrite and NO have definitively entered the molybdenum world and opened a series of exciting questions regarding the chemistry of these ancient molybdenum centres.

## Abbreviations

AnAmmOx, anaerobic ammonium oxidation; AO, aldehyde oxidase; AOR, aldehyde oxidoreductase; Cb, cytoglobin; Cc, cytochrome *c*; CcO, Cc oxidase; ComAmmOx, complete ammonium oxidation; DMSO, dimethylsulfoxide; DMSOR, dimethylsulfoxide reductase; EPR, electron paramagnetic resonance spectroscopy; Hb, haemoglobin; mARC, mitochondrial amidoxime reducing component; Mb, myoglobin; (MGD)_2_-Fe, iron-*N*-methyl-D-glucamine dithiocarbamate; (MGD)_2_-Fe-NO, mononitrosyl-iron-(MGD)_2_-Fe complex; NaR, nitrate redictase; Nb, neuroglobin; NO, nitric oxide; NOS, NO synthase; OAT, oxygen atom transfer; OAT-A, oxygen atom abstraction; OAT-I, oxygen atom insertion; SO, sulfite oxidase; SOD, superoxide dismutase; XD, xanthine dehydrognease; XO, xanthine oxidase.
